# Large gap electron-hole superfluidity and shape resonances in coupled graphene nanoribbons

**DOI:** 10.1038/srep24860

**Published:** 2016-04-25

**Authors:** M. Zarenia, A. Perali, F. M. Peeters, D. Neilson

**Affiliations:** 1 Department of Physics, University of Antwerp, Groenenborgerlaan 171, B-2020 Antwerpen, Belgium; 2 Dipartimenti di Fisica e di Farmacia, Università di Camerino, 62032 Camerino, Italy

## Abstract

We predict enhanced electron-hole superfluidity in two coupled electron-hole armchair-edge terminated graphene nanoribbons separated by a thin insulating barrier. In contrast to graphene monolayers, the multiple subbands of the nanoribbons are parabolic at low energy with a gap between the conduction and valence bands, and with lifted valley degeneracy. These properties make screening of the electron-hole interaction much weaker than for coupled electron-hole monolayers, thus boosting the pairing strength and enhancing the superfluid properties. The pairing strength is further boosted by the quasi one-dimensional quantum confinement of the carriers, as well as by the large density of states near the bottom of each subband. The latter magnifies superfluid shape resonances caused by the quantum confinement. Several superfluid partial condensates are present for finite-width nanoribbons with multiple subbands. We find that superfluidity is predominately in the strongly-coupled BEC and BCS-BEC crossover regimes, with large superfluid gaps up to 100 meV and beyond. When the gaps exceed the subband spacing, there is significant mixing of the subbands, a rounding of the shape resonances, and a resulting reduction in the one-dimensional nature of the system.

Superfluidity of spatially separated electrons and holes was predicted nearly half a century ago^1^ but up to now experimental observation of this exotic state has been elusive at zero magnetic field, notwithstanding multiple attempts on very different systems. The discovery of the wonder material graphene in conjunction with the large band gap insulator hexagonal boron nitride (h-BN) has raised new hopes for realization of this new collective many body state. Recently, superconductivity at temperatures above liquid Helium has been reported in doped monolayer graphene by four groups, amplifying interest in quantum coherent phenomena in graphene^2^.

Monolayer graphene is an atomically flat, gapless semiconductor with near identical conduction and valence bands. Spatially separated electron-doped and hole-doped monolayers can be completely insulated from each other with just a few atomic layers of h-BN^3^,^4^. With such small spatial separations, electron-hole pairing by direct Coulomb attraction is expected to be strong^5^,^6^,^7^. However the linear dispersion of the monolayer graphene energy bands results in very strong Coulomb screening of the electron-hole pairing attraction, and this suppresses superfluidity in coupled electron-hole graphene monolayers^3^,^8^. To overcome the strong screening, refs 9 and 10 proposed using coupled electron-hole graphene multilayers. Using multilayers takes advantage of the nonlinear dispersion of their energy bands^11^,^12^, and the existence of a gap between the conduction and valence bands when a gate potential is applied.

Here we propose a new design to boost electron-hole pairing and the onset of superfluidity using nanoribbons etched in monolayer graphene. Monolayer sheets of graphene are promising candidates for applications in transparent conductive films, electronic and opto-electronic devices, actuators, sensors, composites, and more. However a serious limitation of graphene monolayers is that field-effect transistor (FET) devices are not possible because the massless nature of the electrons prevents electron confinement in graphene. Quasi-one-dimensional graphene nanoribbons with tuneable band gaps resolve this issue, with important implications for the fabrication of novel and ultrafast electronic nanodevices. For example, FET devices with 100 GHz switching frequencies have been fabricated using graphene nanoribbons^13^. The nanoribbon edges can be terminated using a variety of different atoms, which opens up application opportunities, in particular for nanoribbons in polymer hosts for fabrication of novel composite materials^14^,^15^. Finally, graphene nanoribbons are showing great promise as electrode materials for batteries and supercapacitors^16^.

The electronic properties of graphene nanoribbons depend on the type of edge termination^17^. We focus on armchair-edge terminated nanoribbons since (i) their subbands are parabolic around their minima (Fig. 1(a)), (ii) there is a sizeable semiconductor-like energy gap between the conduction and valence bands, and (iii) the valley degeneracy of monolayer graphene is lifted. These properties combine to greatly reduce the strength of screening of the electron-hole pairing interaction. Note that uniform armchair graphene nanoribbons of widths 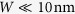 have recently been fabricated^18^.

Figure 2 shows the device we are proposing. It consists of two armchair-edge terminated monolayer graphene nanoribbons, one electron-doped and the other hole-doped, separated by a few atomic layers of a h-BN insulating barrier. The nanoribbons are independently contacted, and top and back metal gates control the carrier densities.

In addition to reducing the effect of screening, electron-hole pairing strengths will be further boosted in our proposed system by the enhanced density of states near the minimum of each subband (see Fig. 1(b)) that arises from the van Hove singularities of the quasi-one-dimensional nanoribbons, and also by the quantum confinement of the carriers in the nanoribbons. Enhancement of superconducting gaps and critical temperature in striped systems due to shape resonances and quantum confinement at the nanoscale was predicted in refs 19, 20, 21. Superconductivity has been observed in quasi-one-dimensional systems including Sn and Al metallic nanowires and carbon nanotubes, with enhanced transition temperatures as compared with their bulk values^22^.

## Methods

**Figure 1:**
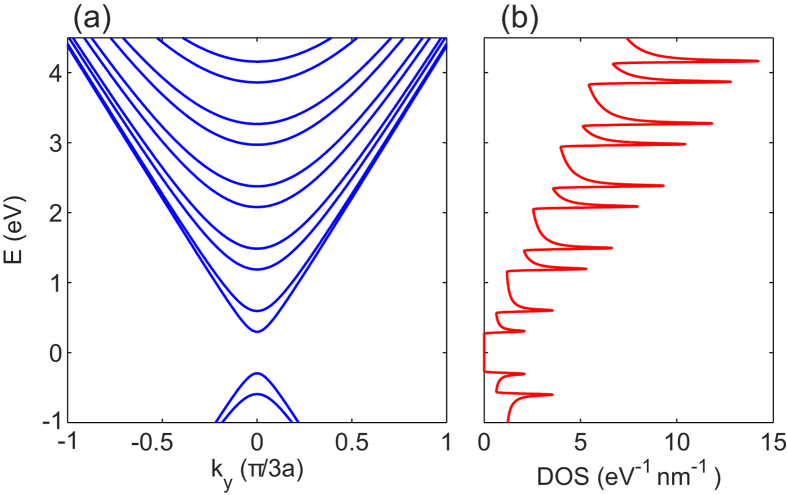
(**a**) Lowest single-particle energy subbands *ε*_*j*_(*k*_*y*_), *j* = 1, 2, … in an armchair graphene nanoribbon of width *W* = 2 nm. (**b**) Corresponding density of states DOS(*E*) in nanoribbon. Van Hove singularities are visible at bottom of each subband.

We take the *y*-direction parallel to the nanoribbons, with the carriers confined in the transverse *x*-direction. Figure 1(a) shows the single-particle energy subbands obtained in the continuum model, 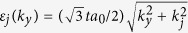, *j* = 1, 2, … for an armchair graphene nanoribbon of width *W* = 2 nm. The intralayer hopping energy *t* = 2.7 eV^23^ and the graphene lattice constant *a*_0_ = 0.24 nm. 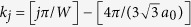 is the quantized wave-number for the *j*-subband in the *x*-direction. Figure 1(b) shows the corresponding density of states DOS(*E*). The van Hove singularities coincide with the bottom of each subband.

Not only the finite width of the nanoribbons but also their multiple occupied subbands make the system only quasi-one-dimensional. In addition, in the superfluid state the energy gap mixes close-by subbands. The quasi-one-dimensionality together with the subband mixing will suppress order parameter fluctuations that are responsible for destroying superfluidity in a pure one-dimensional system. For these reasons we can calculate properties of the superfluid ground state using mean field theory.

Recently ref. 24 discussed a quasi-condensate of excitons in coupled electron-hole one-dimensional wires using the weak-coupled BCS gap equation in the low density limit with only the lowest subband contributing to the pairing, and with screening neglected. Since only one channel was considered, there are no shape resonance effects. Also, because of the one dimensionality, fluctuations of the order parameter should be severe and would strongly suppress superfluidity. Interestingly, ref. 24 argues that even in the one-channel case, the finite size of the nanoribbons would allow for short range superfluid correlations. In our case the many available channels due to the multiple subbands involved in the pairing allow for a suppression of the critical fluctuations, which would favour the observation of conventional long-range superfluidity.

Our calculations are for coupled electron-hole armchair graphene nanoribbons of equal width *W* and equal (two-dimensional) electron and hole densities *n* = (*r*_0_*W*)^−1^, where *r*_0_ is the average inter-particle spacing along the nanoribbon. The subbands *ε*_*j*_(*k*_*y*_), *j* = 1, 2, … are identical for the electrons and holes.

Because of the multiple subband structure, the zero temperature mean field equations for the superfluid state acquire an additional index for the subband *j*. The equations for the wave-vector dependent superfluid energy gaps Δ_*j*_(*k*_*y*_) for subbands *j* become,


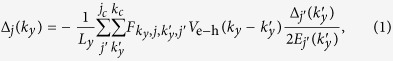


where *L*_*y*_ is the nanoribbon length, 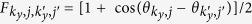 is the form factor^8^ coming from the overlap of the single-particle nanoribbon wave functions, with 
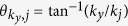
, *V*_e−h_(*q*) is the effective electron-hole pairing interaction, and 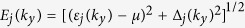 is the single-particle energy dispersion in the superfluid state for subband *j*. The wave-vector 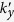 is bounded by the Brillouin zone boundary ±*k*_*c*_. We truncate the sum over the subband index at *j*′ = *j*_*c*_, where *j*_*c*_ is the lowest subband with a minimum above the graphene nanoribbon work function energy, taken to be ~4.5 eV. The chemical potential *μ* is fixed by the density equation,


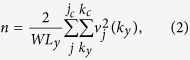


where 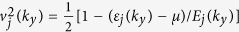, and the prefactor 2 is the spin degeneracy. There is no valley degeneracy for armchair graphene nanoribbons.

We neglect screening in the calculations for the following reasons. Screening is expected to be weak when the superfluid state lies in the strongly-coupled BEC or BCS-BEC crossover regimes because the superfluid gap in these regimes of pairing is comparable to the Fermi energy, resulting in a large smearing of the Fermi surface, and electron-hole pairs that are compact compared with their average spacing. This makes their mutual interactions dipolar and weakly repulsive. Similar arguments suggest in the BEC regime that electron-electron interactions between pairs will be weakened by the compensatory electron-hole interactions^25^, and so we also neglect electron-electron interactions. The expected suppression of screening is confirmed by the following numerical calculations.

In the superfluid state, the RPA-like static screening polarization bubble for small *qd* that is responsible for screening the electron-hole pairing interaction is given by the sum,


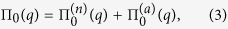


where 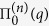 and 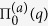 are the normal and anomalous polarizabilities in the superfluid state. These are constructed from pairs of the normal and anomalous Green functions of BCS theory, respectively. The resulting expressions are^9^,


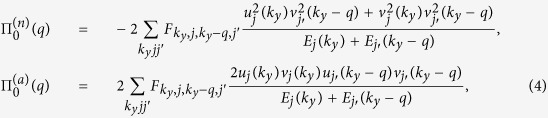


where 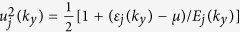. For convenience we write *q*_*y*_ as *q*.

**Figure 3:**
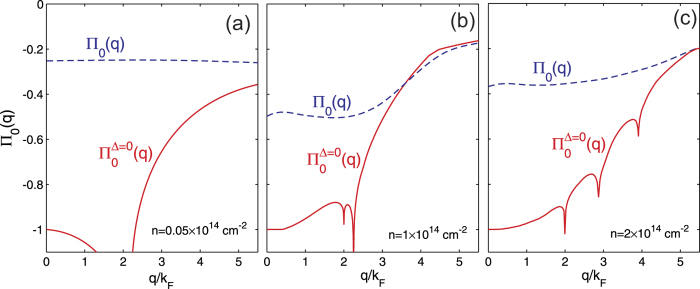
The polarization function ∏_0_(*q*) in the superfluid state for carrier densities *n* as labelled, compared with the corresponding Lindhard polarization function for the normal state of the multi-subband graphene nanoribbon, 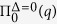 of width *W* = 2 nm and separation *d* = 3 nm. The functions are normalized to 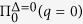.

The opening of the superfluid energy gap Δ at the Fermi surface exponentially suppresses particle-hole processes with energies less than Δ, and it is precisely these low-energy processes that screen the bare interaction in the normal state. As well as a suppression of the screening polarization bubble with diagonal Green functions, there is an additional cancelling contribution from the anomalous polarization bubble with off-diagonal Green functions^8^. Figure 3 compares the polarization function ∏_0_(*q*) in the superfluid state with the corresponding Lindhard polarization function for the normal state of a multi-subband graphene nanoribbon of width *W* = 2 nm and separation *d* = 3 nm. The figure shows that the presence of the superfluid gap strongly suppresses ∏_0_(*q*) for 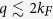. It is at small momentum transfers *q* where the unscreened electron-hole pairing interaction is peaked, and hence this suppression of screening can lead to strong pairing. For this reason we are able, to a good approximation, to neglect screening effects in the pairing interaction.

The results of Fig. 3 contain an additional interesting new phenomenon, that of a competition between superfluidity and Coulomb screening at finite momenta. For each 1D subband, the Fermi surface consists of just two discrete points *k* = ±(*k*_*F*_)_*i*_. A consequence of this is that the static charge response in the normal state (Δ = 0) diverges logarithmically when the transfer momentum *q* connects the two Fermi points, *q* = 2(*k*_*F*_)_*i*_ (the perfect nesting condition). The polarization bubble in the normal state will have multiple divergences associated with subbands successively crossing the Fermi level. This is seen in the three panels of Fig. 3, and is a geometrical effect typical of 1D that in the DOS leads to the multiple Van Hove singularities shown in Fig. 1(b). When superfluid gaps open in the single-particle excitation spectrum, there are no longer zero energy states available for charge excitations, and the singularities in the polarization bubble will be suppressed. The multiple peaks in the normal state polarization bubble will be significantly rounded by a sizeable smoothing of the momentum distribution that is induced by the large superfluid gaps. This rounding of the peaks is clearly seen in the results in Fig. 3. Interestingly, the superfluid state with large gaps shields the system against a Peierls instability typical of 1D materials. This is primarily due to the fact that the perfect nesting condition is no longer realizable in the presence at low energies of sufficiently large pairing gaps.

It should be noted, however, in the weak-coupled BCS regime where the superfluid gap is small compared with *E*_*F*_, that screening becomes a strong effect, and in this regime screening suppresses the superfluidity. Thus we expect the unscreened approximation will break down for densities at which it predicts BCS superfluidity, and that the system will in fact remain in the normal state for those densities^9^,^26^. For this reason, we present results only in the BEC and crossover regimes.

In Eq. (1) we take the effective electron-hole pairing attraction *V*_e−h_(*q*) to be the bare Coulomb interaction. For electrons and holes confined in nanoribbons of width *W*, separated by an insulating h-BN barrier of thickness *d* and dielectric constant *κ* = 3, we obtain^27^,


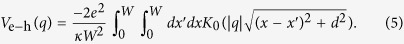


One should in principle include in *V*_e−h_(*q*), contributions from all inter-subband and intra-subband electron-hole scatterings, *v*_*ijmn*_(*q*), where *i*, *j*, *m*, *n* are the subband indices. However Fig. 1 of ref. 27, which plots *v*_*ijmn*_(*q*) for armchair graphene nanoribbons, shows that the intra-subband electron-hole scatterings, with *i* − *j* = *m* − *n* = 0, dominate over all inter-subband scattering terms. In the small *q* limit, it is only the intra-subband scatterings that logarithmically diverge. For this reason, in Eq. (1) we may limit ourselves to the intra-subband contributions to the pairing interaction.

## Results

**Figure 4:**
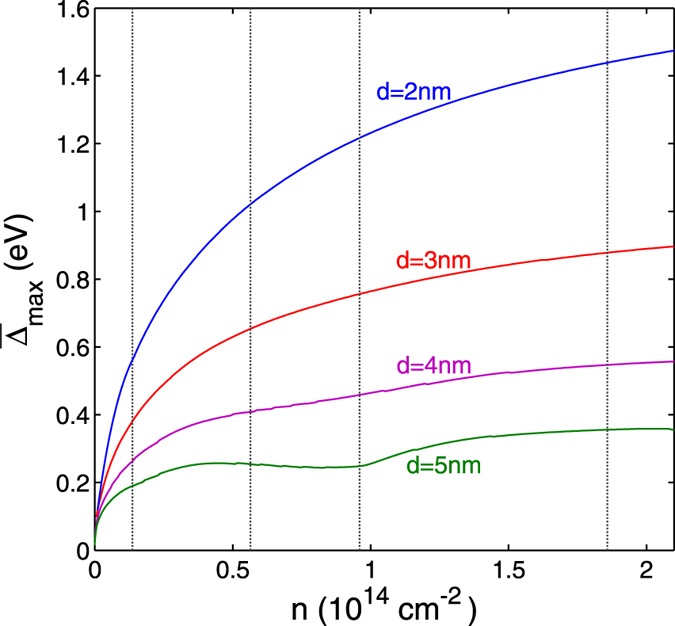
Maximum superfluid gap 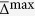 averaged over the subbands for different thicknesses *d* of the insulating barrier separating the nanoribbons. Nanoribbon width is *W* = 2 nm. Densities at which *E*_*F*_ enters the bottom of a new subband *ε*_*j*_ are indicated by the vertical dotted lines.

Figure 4 shows the maximum superfluid gap 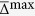 as a function of the density *n*, averaged over the multiple subbands of the nanoribbons, calculated using Eqs (1, 2, 3, 4, 5). 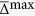 is the maximum value of the wave-vector dependent 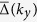 averaged with respect to the subband index *j*. The nanoribbon width is *W* = 2 nm, and *d* is the thickness of the insulating barrier. The densities at which the Fermi energy enters the bottom of a new subband *ε*_*j*_, *j* = 1, 2, …, are indicated by the vertical lines. The superfluid gap 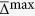 is of the order of eV. It is interesting to note that in the low-density regime, 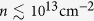, the maximum superfluid gap lies in the same range 200–20 meV predicted by Mohammadzadeh *et al.*^28^ for the binding energy of excitons within a single nanoribbon. 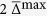 is a good estimate of the exciton binding energy when 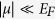. We note that the separation of the nanoribbons in our system is much less than the effective Bohr radius.

In Fig. 4 we notice a local boost in 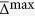 near the minimum of each subband for barrier thickness *d* = 5 nm. This boost arises from shape resonance effects associated with the van Hove singularities (Fig. 1(b)) and the quantum size effects in the pairing interaction. However, for thinner barriers 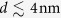, where the electron-hole pairing becomes progressively stronger, the shape resonance effects are masked by a mixing of the subbands caused by the large superfluid gap. As 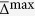 grows larger than the typical spacing between subbands, the system becomes decreasing less one-dimensional in character, thanks to the many channels available both for Cooper pairing and for forming the superfluid condensate.

**Figure 5:**
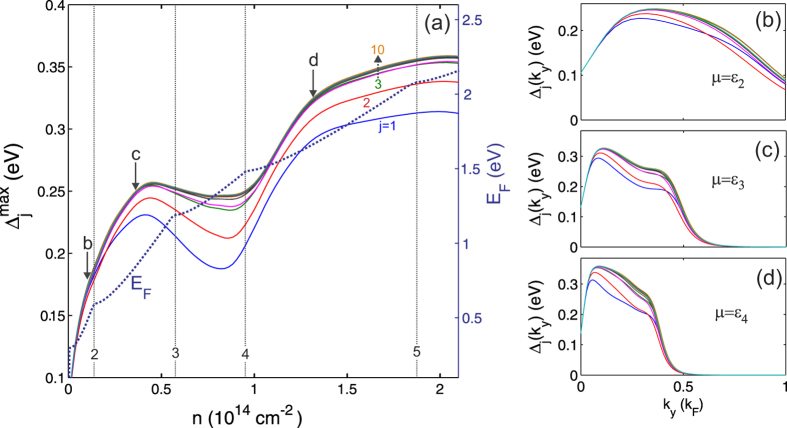
(**a**) Maximum superfluid gap 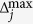 for subbands *j* = 1, 2 … as function of density *n*. The dotted line shows the total Fermi energy *E*_*F*_. Nanoribbon widths *W* = 2 nm and barrier thickness *d* = 5 nm. The densities at which *E*_*F*_ enters the bottom of a new subband are indicated by the vertical lines. Note that the 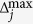 are all of order *E*_*F*_. Right panels (**b**–**d**): momentum-dependent gaps Δ_*j*_(*k*_*y*_) for subbands *j* at densities marked by the arrows in panel (**a**) (at which *μ* = *ε*_2_, *ε*_3_, *ε*_4_).

Figure 5(a) shows the maximum superfluid gap 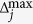 for the separate subbands *j* as a function of *n* for nanoribbon width *W* = 2 nm and barrier thickness *d* = 5 nm. For comparison, the total Fermi energy *E*_*F*_ of the non-interacting nanoribbon system at density *n* is also shown. The vertical lines mark the densities at which *E*_*F*_ enters a new subband. For the lowest conduction subbands, there is a notable local boost in 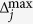 as *E*_*F*_ enters a new subband. This boost takes the form of a shape resonance in the superfluid gaps associated with a particular subband. However even for the lowest subbands, we lose some fine structure of the shape resonances because the gap mixes close subbands. Over the density range shown, the 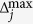 remain always of order *E*_*F*_, and hence they lie in the strongly coupled regime.

Figure 5(b–d), show the momentum-dependence of the subband gaps Δ_*j*_(*k*_*y*_) for densities at which the chemical potential *μ* enters a new low-lying subband (marked in Fig. 5(a) by the vertical arrows). The peaks in Δ_*j*_(*k*_*y*_) are broad on the scale of *k*_*F*_ = *π*/(2*r*_0_), the inverse of the average inter-particle spacing, which confirms that we are in the BCS-BEC crossover regime of compact electron-hole pairs. In panels (c) and (d) of Fig. 5, the multiple peaks of Δ_*j*_(*k*_*y*_) are associated with the different Fermi energies of the subbands (*k*_*F*_)_*j*_, displaying a remaining fermionic character of the Cooper pairing in the BCS-BEC crossover regime.

**Figure 6:**
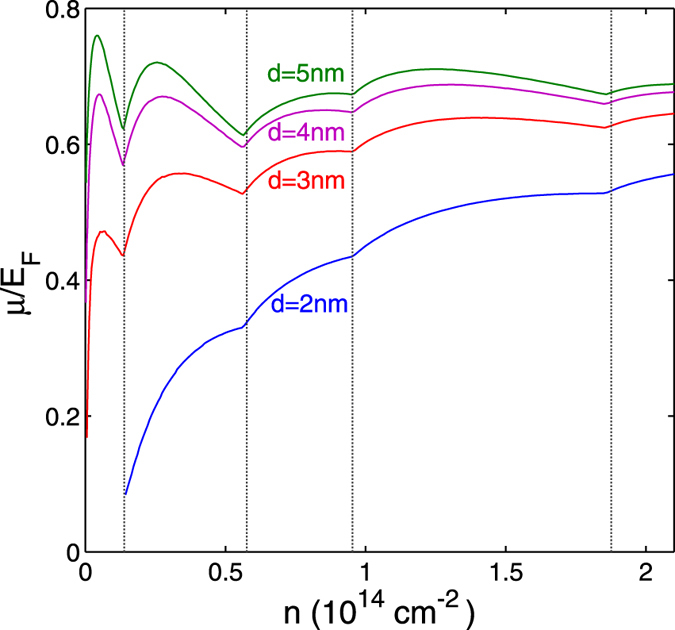
Ratio of chemical potential *μ* to Fermi energy *E*_*F*_ as function of density *n* for different values of the thickness *d* of the insulating barrier separating the nanoribbons. Nanoribbon width *W* = 2 nm. The vertical dotted lines show the densities at which *E*_*F*_ enters the bottom of a subband.

Figure 6 shows the chemical potential *μ* as a function of density *n* for nanoribbon width *W* = 2 nm and barrier thickness *d*. The *μ* is normalized to the corresponding Fermi energy of the non-interacting nanoribbon system at density *n*. The chemical potential is strongly renormalized with respect to the Fermi energy over the full range of *n* and *d* shown. When *E*_*F*_ enters a subband, *μ* has a dip. This is in contrast to the peak seen in the superfluid gap, and it is a shape antiresonance caused by the shape-resonance-generated peak in the gap. As *d* increases the pairing strength weakens, *μ* increases towards *E*_*F*_ and the shape (anti)resonances become sizeable, indicating that the system has entered the BCS-BEC crossover regime. This is particularly evident in Fig. 6 for *d* = 4 − 5 nm. In the case of *d* = 2 nm, 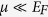 and the shape (anti)resonances are completely smoothed out. This is a result of large superfluid gaps and it signals that the system is in the strong pairing BEC regime. When the density increases, the system always evolves towards the weaker pairing BCS regime for all values of *d*, with *μ* eventually arriving at *E*_*F*_.

The average pair size of the Cooper pairs *ξ*_*j*_ in subband *j* is defined as the expectation value of the square of the relative coordinate of the Cooper pairs with respect to the square of the BCS wave function projected in the subband. This definition was originally introduced in ref. 29 to investigate the different regimes of pairing in high-*T*_*c*_ superconductivity in cuprates as a function of density. It has been extended to a multigap superconductor throughout the BCS-BEC crossover in ref. 30 and to a multigap quasi-one-dimensional superfluid of ultracold fermions confined in cigar-shaped traps^31^. In wave-vector space,


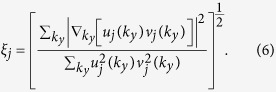


**Figure 7:**
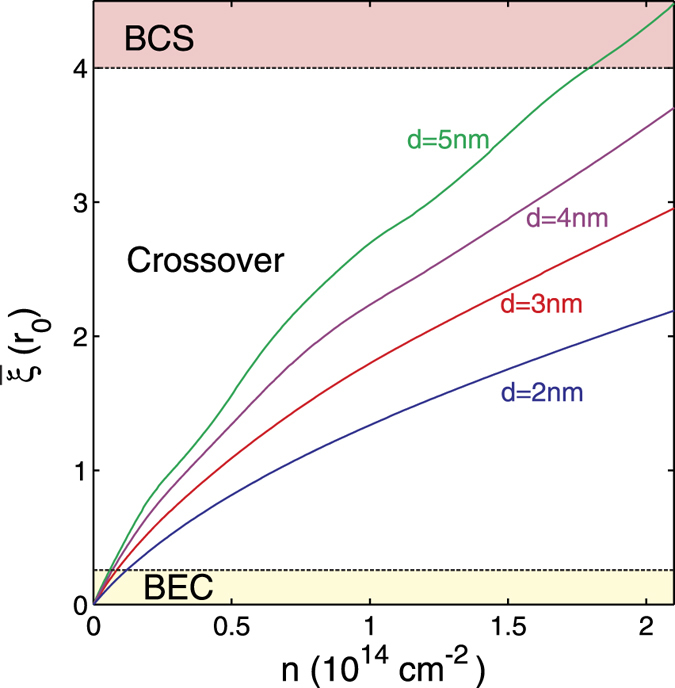
Pair correlation length 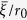 averaged over subbands as function of *n*, the carrier density for different values of the thickness *d* of the insulating barrier separating the nanoribbons. Nanoribbon width *W* = 2 nm.

Figure 7 shows the pair correlation length 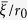 as a function of density normalized to the average inter-particle distance *r*_0_. 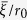 is the partial average pair size for each subband averaged over the subbands. The nanoribbon width *W* = 2 nm. We designate 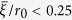 as the BEC regime, 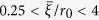 the BCS-BEC crossover regime, and 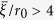 the BCS regime. As discussed, our approximation of neglecting screening is a good one for densities lying in the BCS-BEC crossover and BEC regimes. As expected, the density range for the strongly-coupled regime contracts with increasing barrier thickness *d* because the pairing becomes weaker.

We have neglected effects from impurities and disorder. We expect these effects to be small since while there is no direct information on impurity and disorder effects in graphene nanoribbons, but based on properties of analogous coupled electron-hole graphene monolayers, charge impurities concentrations up to *n*_*i*_ < *k*_*F*_/(*πd*) are not expected to destroy superfluidity^32^. At graphene-hBN interfaces, the charge impurity density *n*_*i*_ ~ 10^10^ cm^−2 ^ (see Ref. 33) so for 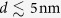, the inequality is satisfied provided 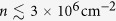. Since at density *n*_*i*_ ~ 10^10^ cm^−2^, the average spacing between charge impurities is orders of magnitude greater than the average spacing of the charge carriers, we do not expect our conclusions on screening to be affected by the presence of such impurities.

## Conclusions

The superfluid gaps in our coupled electron-hole nanoribbon systems are large in absolute value and comparable to the Fermi energy. Both the quasi-one-dimensional confinement and the superfluid shape resonances due to quantum size effects play an important role here. The van Hove singularities in the density of states act non-linearly through the gap equation to significantly enhance the magnitude of the superfluid gaps. In the range of nanoribbon densities and barrier separations considered, we find that the electron-hole superfluid is for the most part in the strongly coupled pairing regime and that screening is strongly suppressed by the large superfluid gaps.

When the superfluid gaps are comparable to the subband energy separations, the gaps mix the subbands and this results in a rounding of the shape resonances. This effect is most pronounced for small separations between the nanoribbons where the electron-hole coupling is particularly strong. For larger separations, the electron-hole coupling is weaker and the superfluid gaps are smaller. This results in weaker subband mixing. When this is the case the shape resonances are sharper, which strengthens the local amplification of the gaps.

In our quasi-one-dimensional system there is no direct link between the superfluid transition temperature and the size of the superfluid gaps calculated within mean field. In our proposed device we find zero temperature superfluid gaps comparable to the Fermi energy, with gaps of order of hundreds of meV. Thus high transition temperature electron-hole superfluidity could be expected, with properties that are tuneable by changing the density. The device configurations we propose are experimentally realizable with current technologies. A superlattice formed of such nanoribbon devices could further stabilize the electron-hole superfluid phase over large areas.

## Additional Information

**How to cite this article**: Zarenia, M. *et al.* Large gap electron-hole superfluidity and shape resonances in coupled graphene nanoribbons. *Sci. Rep.* **6**, 24860; doi: 10.1038/srep24860 (2016).

## Figures and Tables

**Figure 2:**
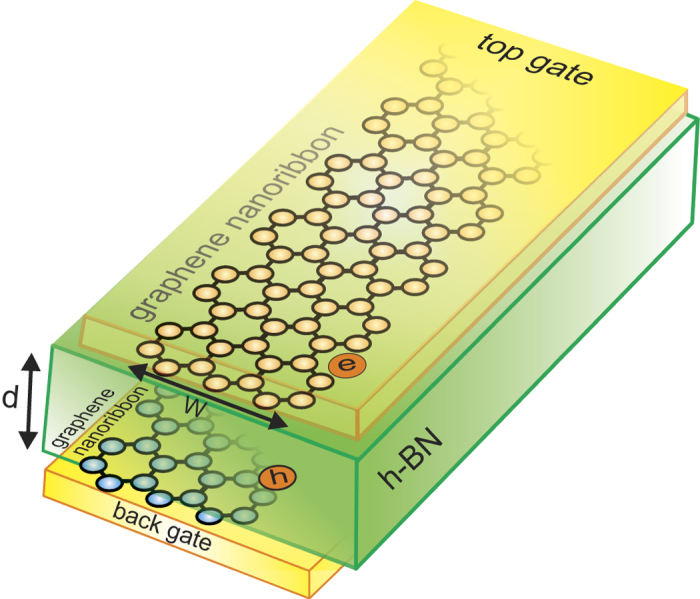
Proposed device. Upper electron-doped and lower hole-doped armchair-edge terminated graphene nanoribbons of widths *W* separated by h-BN insulator of thickness *d*. Top and back gates control electron and hole densities. Gates are separated from nanoribbons by h-BN layers. Nanoribbons are independently contacted.
